# Methylomics analysis identifies a putative STAT3 target, *SPG20*, as a noninvasive epigenetic biomarker for early detection of gastric cancer

**DOI:** 10.1371/journal.pone.0218338

**Published:** 2019-06-13

**Authors:** Kuo-Liang Wei, Jian-Liang Chou, Yin-Chen Chen, Hongchuan Jin, Yu-Min Chuang, Cheng-Shyong Wu, Michael W. Y. Chan

**Affiliations:** 1 Division of Gastroenterology, Chang Gung Memorial Hospital, Chia-Yi, Taiwan; 2 Laboratory of Cancer Biology, Key Lab of Biotherapy in Zhejiang, Sir Run Run Shaw Hospital, Medical School of Zhejiang University, Hangzhou, China; 3 Department Biomedical Sciences, National Chung Cheng University, Chia Yi, Taiwan; 4 Center for Innovative Research on Aging Society, National Chung Cheng University, Chia Yi, Taiwan; 5 Center for Nano Bio-Detection, National Chung Cheng University, Chia Yi, Taiwan; 6 Epigenomics and Human Disease Research Center, National Chung Cheng University, Chia Yi, Taiwan; Sapporo Ika Daigaku, JAPAN

## Abstract

Gastric cancer is a leading cause of cancer worldwide. Our previous studies showed that aberrant activation of JAK/STAT3 signaling confer epigenetically silences STAT3 target genes in gastric cancer. To further investigate the clinical significance of this phenomenon, we performed Illumina 850K methylation microarray analysis in AGS gastric cancer cells, and cells depleted of STAT3. Integrative computational analysis identified *SPG20* as a putative STAT3 epigenetic target, showing promoter hypomethylation in STAT3-depleted AGS cells. Bisulphite pyrosequencing and qRT-PCR confirmed that *SPG20* is epigenetically silenced by promoter hypermethylation in a panel of gastric cancer cell lines including AGS cells, but not in immortalized gastric epithelial GES cells. Expression of *SPG20* could be restored by the treatment with a DNMT inhibitor, further suggesting that *SPG20* is epigenetically silenced by promoter methylation. Clinically, a progressive increase in *SPG20* methylation was observed in tissues samples from gastritis (n = 34), to intestinal metaplasia (IM, n = 33), to gastric cancer (n = 53). Importantly, *SPG20* methylation could be detected in cell-free DNA isolated from serum samples of gastritis, IM and gastric cancer patients, having a progressive similar to tissues. Taken together, *SPG20*, a potential STAT3 target, is frequently methylated in gastric cancer, representing a novel noninvasive biomarker for early detection of this deadly disease.

## Introduction

Gastric cancer is a leading cause of cancer deaths worldwide [1]. Despite advances in cancer therapy, gastric cancer patients still have a poor 5-year survival of less than 15% [2], likely due to a lack of biomarkers for early detection. Although endoscopic screening for early gastric cancer may improve patient’s survival, noninvasive, sensitive, and specific population screening assays are currently not available and are therefore urgently needed.

Epigenetic modifications, including DNA methylation, play an important role in transcriptional regulation, and embryonic and disease development [3, 4]. Due to its stability, methylation at the cytosine of CG dinucleotides has been found both in tissues and bodily fluids such as plasma and serum. These attributes make DNA methylation an attractive target for non-invasive cancer detection in cell-free DNA (cfDNA, also known as liquid biopsy) [5, 6].

Our laboratory has long been dedicated to the development of DNA methylation as a biomarker for disease monitoring [7–9]. Additionally, we previously demonstrated that DNA methylation could be detected in bodily fluids, in several human diseases, including cancers [10, 11]. However, promising DNA methylation biomarkers, for noninvasive early detection of gastric cancer, remain lacking.

Our previous studies demonstrated that aberrant activation of JAK/STAT signaling, triggered by *H*. *pylori* infection, can lead to epigenetic silencing of tumor suppressors in gastric cancer [12, 13]. In this study, we examine additional STAT3 targets that are epigenetically silenced by DNA methylation in gastric cancer, representing sensitive biomarkers for noninvasive detection of gastric cancer.

## Materials and methods

### Patient samples

Patient samples (biopsy and serum) were obtained from Chang Gung Memorial Hospital, Chiayi, Taiwan or the Medical School of Zhejiang University, Hangzhou, China from March 2013 to February 2016 (Table 1). All samples were stored at -80°C before subsequent processing for analysis. All human subject assessments were approved by the Institutional Review Board (IRB) of the Chang Gung Memorial Hospital, Chiayi, Taiwan and the ethics committee of Zhejiang University, Hangzhou, China. The study was carried out in strict accordance with approved guidelines. Written informed consent was obtained from all participants.

**Table 1:** Summary of clinico-pathological data of patients samples.

	Gastric cancer (n = 53)	Non-cancer (n = 107)
Age Median Range	69 43~87	57 25~87
Sex Male Female	37 16	60 47
Disease type normal gastritis intestinal metaplasia(IM) cancer	53	40 34 33
*H*. *pylori* infection positive negative	12 28	40 55
Median survival	8.2 (n = 17)	
Stage (for cancer) I II III IV	12 10 20 12	
Metastasis (for cancer) Yes No	21 32	

### Cell culture

Gastric cancer cell line (AGS, KATO III, MKN28, MKN45, SNU1, SNU16, purchased from ATCC, Manassas, VA) and an immortalized gastric epithelial cell line, GES (a kind gift from Dr. Jun Yu, The Chinese University of Hong Kong, Hong Kong) were maintained in RPMI 1640 (Gibco, Waltham, MA) supplemented with 10% FBS (Gibco) and 1% Penicillin-Streptomycin (Gibco). All cells were maintained at 37°C, with 5% CO_2,_ under a humidified incubator. Cells were treated with 0.5μM 5-aza-2’-deoxycytidine (5-azaDC, Sigma, St. Louis, MO) for 3 days and harvested for RNA extraction.

### DNA extraction, RNA extraction, and quantitative reverse transcription-PCR

DNA was extracted using a Genomic DNA Mini Kit (Geneaid, Taiwan), according to the manufacturer’s instructions. DNA was then eluted in 50μl distilled water and stored at -20°C until use. Cell-free DNA was extracted from 500ml serum samples using QIAamp DNA Blood Mini Kit (Qiagen GmbH, Germany). RNA was extracted using TriZol reagent (Invitrogen, Carlsbad, CA), according to the manufacturer’s protocol. To remove potential contaminating DNA from the complementary DNA, 1μg of total RNA was treated with DNase I (Amplification Grade, Invitrogen), prior to reverse transcription (Superscript ll RT, Invitrogen). Real-time PCR reactions were performed on an ABI Step-One real-time PCR system (Applied Biosystems, Foster City, CA) with specific primers (Table 2). Relative gene expression was determined by comparing the threshold cycle (Ct) of the test gene against the Ct value of GAPDH, in a given sample. (i.e., the comparative Ct method).

**Table 2:** Primer sequence used in this study.

	Sequence 5’-3’
**RT-PCR**	
GAPDH forward GAPDH reverse LPHN3 forward LPHN3 reverse SOX5 forward SOX5 reverse ABR forward ABR reverse *SPG20* forward *SPG20* reverse	CCCCTTCATTGACCTCAACTACAT CGCTCCTGGAAGATGGTGA GAAATATTCTTTGGATTTTGGACC TAGCTCAGAGTCAAGGTGAATTG GGGAACAACAGGTGCTTGAT CTCCACTCAGATTGAAATCCATC AGCGCCTGAAGAAGAAGATGTT TGAATTGCTTCTCTCCACTCTGA AACTAGACCCTCCTCTGACCAA TTTCTTCTGGAACTGGCTCA
**Pyrosequencing**	
*SPG20* forward *SPG20* reverse *SPG20* sequencing	AAGTAATAGAAGATTGTATTTATATGGAATTATTTTTTGA ACAAATTAATCTAATACCTATTTAACCCTTTACCA GGAAGTTTTTTTGTAATGTG
**Methylation-Specific PCR**	
Methylated *SPG20* MF *SPG20* MR Unmethylated *SPG20* UF *SPG20* UR	ATTTTTTGATTCGTAGTTTTTTATAATC AAAAATAAAACCGACCCGA GAATTATTTTTTGATTTGTAGTTTTT AAAATAAAACCAACCCAAAA

### Bisulphite conversion and pyrosequencing

0.5μg genomic DNA was bisulphite modified using an EZ DNA Methylation Kit (Zymo Research, Orange, CA) according to the manufacturer’s protocol. The bisulphite modified DNA was then subjected to PCR amplification, using a tailed reverse primer, including a biotin-labeled universal primer, PCR, and sequencing primers were designed using PyroMark assay Design 2.0 software (Qiagen). The *SPG20* transcription start site (+1094 to +1387) was PCR amplified using specific primer (Table 3), and pyrosequencing was performed using PyroMark Q24 (Qiagen) Pyro Gold Reagents (Qiagen), according to the manufacturer’s protocol. Methylation percentage of six CpG sites located from +1209 to +1229 bp was determined by the fluorescence intensity of cytosines and thymines at each CpG site. *In vitro* methylated DNA (IVD, Merck Millipore, MA) was included as positive control for bisulphite pyrosequencing.

**Table 3:** Association between clinical parameters and *SPG20* methylation in 53 gastric cancer patient samples.

	*SPG20* methylation (n)	P
Age ≥60 <60	24.40 ± 11.81, (43) 24.27 ± 11.93, (10)	0.48
Sex Male Female	24.27 ± 11.93, (37) 24.40 ± 11.81, (16)	0.48
*H*. *pylori* infection Yes No	25.97 ± 12.52, (12) 24.33 ± 11.82, (27)	0.27
Stage High stage (I ~ II) Low stage (III ~ IV)	24.27 ± 11.93, (28) 24.40 ± 11.81, (23)	0.48
Metastasis Yes No	24.27 ± 11.93, (20) 24.70 ± 11.72, (30)	0.43

### Methylation-specific PCR (MSP)

Bisulphite-modified DNA was subjected to MSP for *SPG20* methylation analysis using specific primers (Table 2). 4μl of bisulphite-converted DNA were amplified in a total volume of 20μl containing 10x PCR Buffer, 0.25mM dNTPs, 2mM MgCl_2_, 0.2μM of each primer and 1.25U of Platinum *Taq* DNA polymerase (Invitrogen) at 95°C for 2 min, followed by 40 cycles of denaturing at 95°C for 30 sec, annealing at 60°C for 30 sec, and extension at 72°C for 30 sec, followed by followed by a final extension step of 72°C for 10 min. *In vitro* methylated DNA (IVD, Merck Millipore) was included as positive control and normal blood (NB) was included as a negative control of MSP. 10μl of PCR products were loaded onto 10% polyacrylamide gels, which were then stained with ethidium bromide, and visualized under UV illumination.

### Infinium microarray DNA methylation analysis

Bisulphite-modified DNA from AGS gastric cancer cells and cells depleted of STAT3 was subject to methylation analysis, using an Illumina 850K methylation microarray. The methylation level of each probe (β-value) was defined by the intensity of the methylated allele (M) / (intensity of the unmethylated allele (U) + the intensity of the methylated allele (M) + 100). The microarray data has been deposited in the Gene Expression Omnibus database (accession number: GSE109541).

### Statistical analysis

Unpaired t-tests were used to compare parameters of the various groups. All statistical calculations were performed using the SPSS statistical package (version 18.0) for Windows (IBM, Chicago, IL, USA). In this study, P values <0.05 were considered statistically significant.

## Results

**Fig 1:**
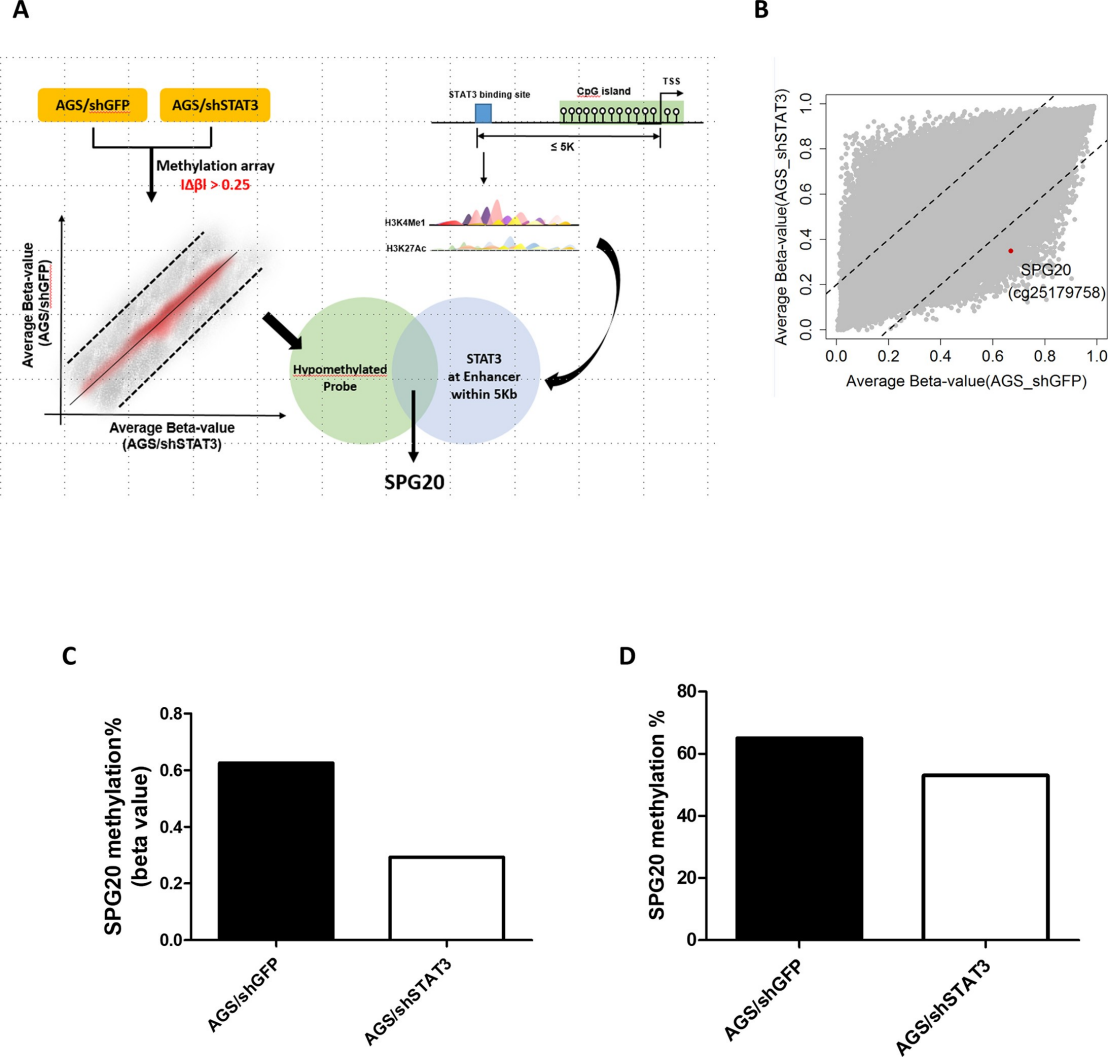
Integrated microarray and bioinformatics analyses identifies *SPG20* as an epigenetically silenced target of STAT3, in gastric cancer. (A) Schematic diagram showing the experimental scheme of this study. Stable transfectants of AGS gastric cancer cells depleted of STAT3 (shSTAT3) or control (shGFP) were subjected to Illumina 850K methylation microarray analysis. Probes showing a changes in β values (|Δβ|) of at least 0.25 (25% methylation difference) were selected. In addition, bioinformatics analysis was performed to identify potential STAT3 targets by filtering genes with at least one STAT3-binding site in potential enhancer region (as enriched with H3K4me1 and H3K27Ac) of the promoter CpG island. One hypomethylated probe and a potential STAT3 target, *SPG20*, was selected for further analysis. (B) Scatter plot showing the average beta-value of each probe in AGS cells depleted of STAT3 or GFP (as control). One of the probes (cg25179758, red dot) showing hypomethylation, located within the *SPG20* promoter region, was selected for further analysis. Methylation% of *SPG20* at the promoter region as determined by (C) methylation microarray (beta value) or (D) bisulphite pyrosequencing.

Our previous studies found that aberrant activation of JAK/STAT signaling could lead to epigenetic silencing of STAT3 targets in gastric cancer [12, 13]. We therefore hypothesized that binding of STAT3 to promoter-proximal CpG islands may affect their methylation status. In this regard, we performed Illumina 850K methylation microarray analysis in bisulphite-treated genomic DNA from AGS gastric cancer cells, and cells depleted of STAT3. Computational predictions were also performed to identify all STAT3-binding sites located in open chromatin regions (as demarcated by H3K4me1 and H3K27Ac) in close proximity to promoter CpG islands (Fig 1A). One probe (cg25179758, Fig 1B and 1C) within the promoter region of *SPG20*, showing differential hypomethylation in STAT3-depleted AGS cells, was identified. Bisulphite pyrosequencing further confirmed that knock-down of STAT3 decreased *SPG20* methylation in AGS cells (Fig 1D).

**Fig 2:**
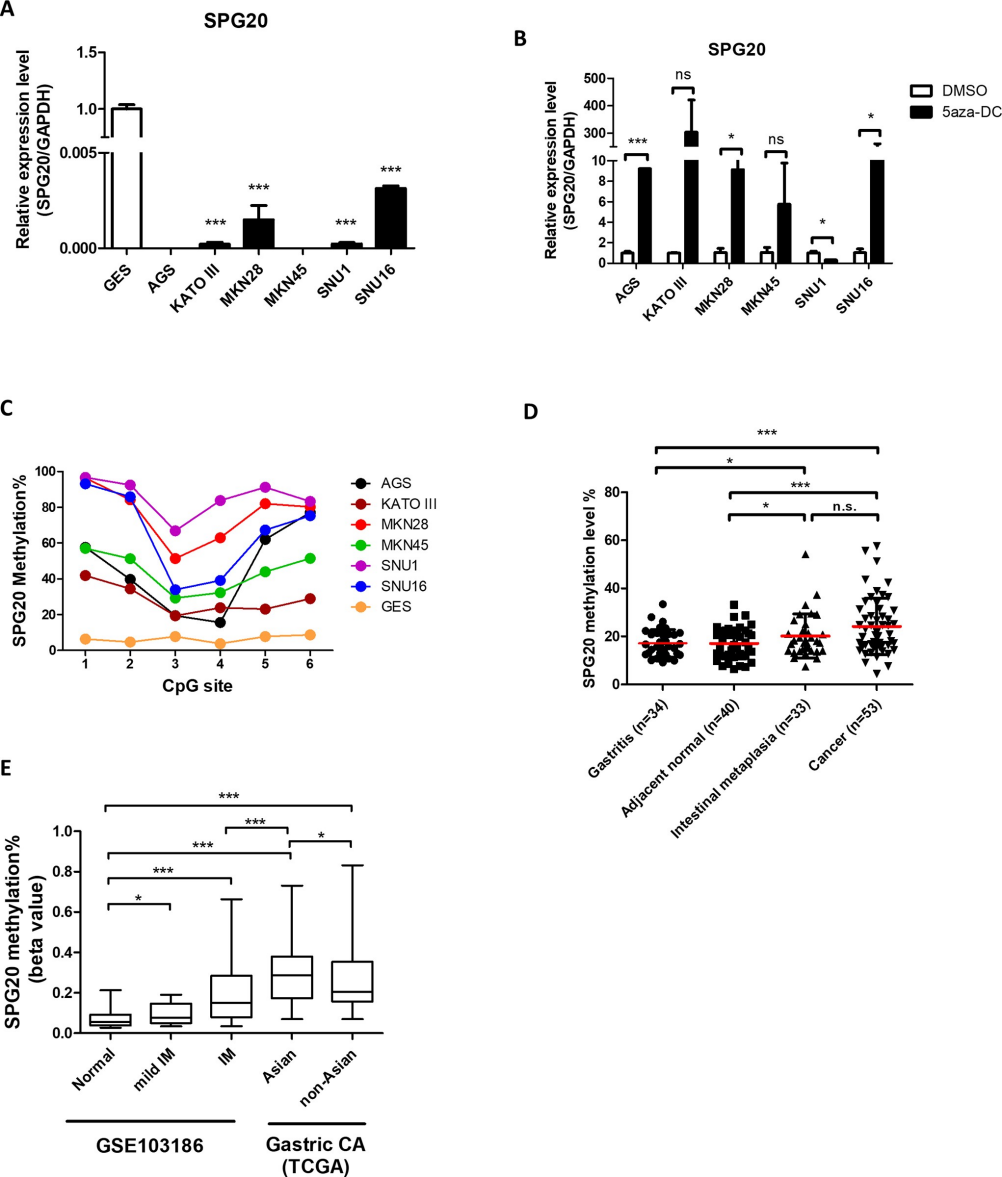
*SPG20* is epigenetically silenced by promoter methylation in gastric cancer. Relative expression of *SPG20* in (A) immortalized GES cells, and a separate panel of gastric cancer cell lines, and (B) 5aza-DC-treated gastric cancer cells, as determined by qRT-PCR. Each bar represents the means ± SD of duplicate experiments. (C) Methylation levels of six CpG sites within the *SPG20* promoter region in GES cells and various gastric cell lines, as determined by bisulphite pyrosequencing. (D) Promoter methylation of *SPG20* in gastritis, tumor adjacent normal, intestinal metaplasia (IM), and gastric cancer tissue samples, as determined by bisulphite pyrosequencing. Red lines denote median values. (E) Promoter methylation of *SPG20* in IM and gastric cancer, as retrieved from GSE103186 (normal and IM) and TCGA (cancer). Significant differences between groups are indicated by *P<0.05, **P<0.01, ***P<0.005, as determined by Mann-Whitney *U*-test.

To further examine the role of DNA methylation in regulating expression of *SPG20*, we assessed its expression in an immortalized gastric epithelial cell (GES), and a panel of gastric cancer cell lines. Except for GES cells, downregulation of *SPG20* was observed in all cancer cell lines (Fig 2A). Treatment with the DNMT inhibitor (5aza-DC) resulted in robust re-expression of *SPG20* in those cells (Fig 2B), due to DNA demethylation of its promoter as confirmed by bisulphite pyrosequencing (Fig 2C).

We then examined the clinical significance of *SPG20* methylation in a cohort of gastritis, intestinal metaplasia (IM), and paired gastric cancer patient samples (Table 1). Although no clinical parameters significantly associated with DNA hypermethylation (Table 3), results from bisulphite pyrosequencing showed higher *SPG20* methylation in gastric tumors, compared to tumor-adjacent normal and gastritis tissues (Fig 2D). Interestingly, IM tissues also showed a higher *SPG20* methylation, compared to adjacent normal and gastritis tissues (Fig 2D).

We also compared *SPG20* methylation in datasets (GSE103186 and TCGA) from normal gastric epithelial, IM, and gastric cancer tissues obtained from two publicly available databases. Consistently, cancer tissues showed higher *SPG20* methylation than normal and IM samples (Fig 2E). As demonstrated in our cohort, samples from IM also showed higher *SPG20* methylation than those from normal tissues. It is interesting to note that *SPG20* methylation, in cancer tissues from Asian populations, were higher than those from non-Asian populations, suggesting population-specific underlying epigenotypes.

**Fig 3:**
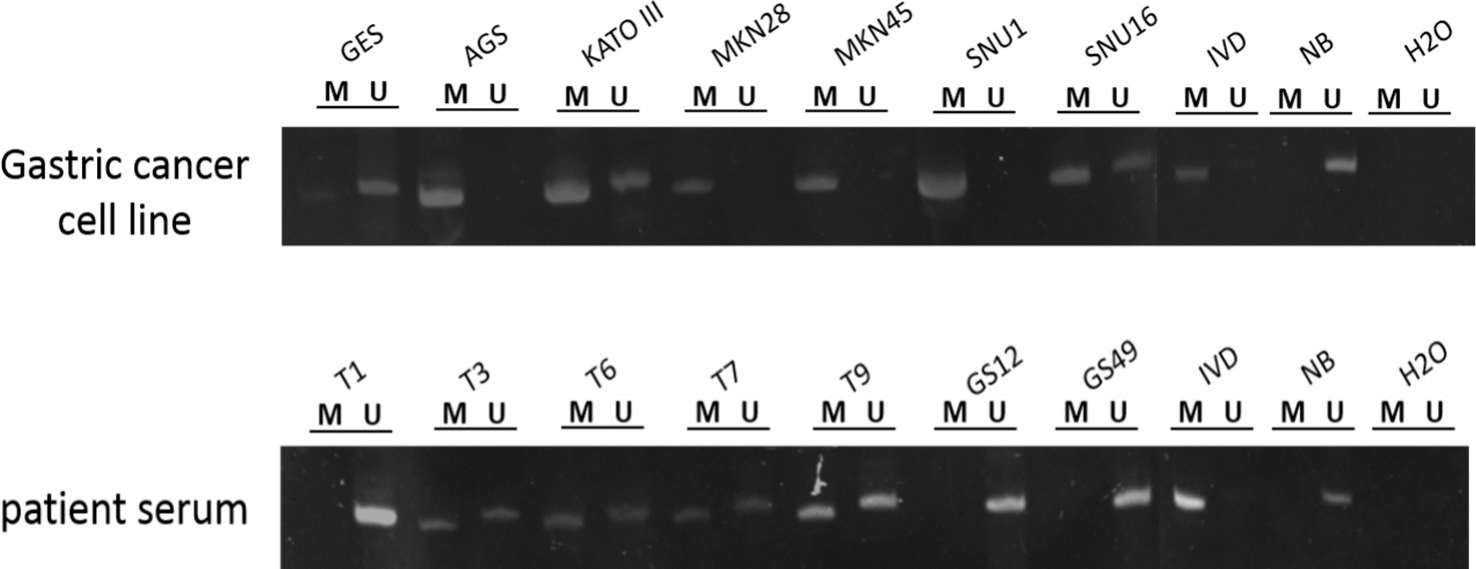
Methylation analysis of *SPG20* in cell lines and cell-free DNA from serum patient samples. Representative gel picture from methylation-specific PCR (MSP) analysis to determine *SPG20* methylation in gastric cancer cell lines (top panel) and patient serum samples (bottom panel). Bisulphite-modified DNA was PCR-amplified using specific primers. “M” and “U” indicate the presence of methylated and unmethylated alleles, respectively. IVD (*in vitro* methylated DNA) was a positive control for methylation and NB (normal blood) was a negative control for methylation. Water (H2O) was used as a negative control for PCR.

To further examine the feasibility of *SPG20* methylation, as a noninvasive methylation biomarker for the early detection of gastric cancer, we performed conventional methylation-specific PCR to amplify short fragment of methylated DNA from cell-free DNA (cfDNA) obtained from serum samples of non-cancer individuals, and patients with IM or gastric cancer. In agreement with our bisulphite pyrosequencing results, *SPG20* methylation could be detected in all gastric cancer cell lines by MSP (Fig 3). Overall, the sensitivity and specificity of gastric cancer detection using *SPG20* methylation, were 88.6% and 75%, respectively (Table 4). Importantly, the sensitivity of cancer or IM detection could remain as high as 87.5% (Table 4).

**Table 4:** Sensitivity and specificity of cancer detection using serum *SPG20* methylation.

Disease type^1^	Cancer only (n = 53)	Cancer or IM (n = 56)
Sensitivity Specificity PPV^2^ NPV^3^	88.6% (47/53) 75.0% (15/20) 90.3% 71.4%	87.5% (49/56) 75.0% (15/20) 90.7% 68.1%

^1^As compared with serum samples from non-cancer (n = 20)

^2^Positive predictive value

^3^Negative predictive value

## Discussion

DNA methylation, an “epigenetic” mode of transcriptional regulation, is altered in numerous pathologies. Consequently, due to its chemical uniqueness and stability (e.g., vs. RNA), disease-associated methylated DNA sequences represent promising tissue and liquid biomarkers. In the current study, by methylation microarray, we identified that a potential STAT3 target, *SPG20*, is differentially methylated in gastric cancer. Cell line studies further confirmed that *SPG20* is epigenetically silenced, by DNA methylation, in gastric cancer cell lines. Clinical studies also demonstrated *SPG20* hypermethylation in tissues and serum samples from IM and gastric cancer patients. Importantly, a progressive increase in *SPG20* methylation, from gastritis to gastric cancer, was observed.

*SPG20*, encoding the multifunctional protein Spartin, has been shown to be involved in several cellular processes. Several studies have found that SPG20, containing a MIT (microtubule interacting and trafficking) domain, is involved in cytokinesis [14]. Cells depleted of SPG20 showed cytokinesis arrest and convoluted midbodies. More recently, SPG20 has been found to be involved in EGFR trafficking and MAPK signaling pathway [15, 16]. Specifically, gastric cancer cells depleted of SPG20 showed increased EGFR expression and phosphorylation of kinase involved in the MAPK signaling. Taken together, these studies suggest that SPG20 may act in early carcinogenesis and proliferation of human cancer. In agreement with those observations, *SPG20* methylation was observed in intestinal metaplasia (IM), an early lesion of gastric cancer, from our own cohort and published data [17]. Although gene silencing of *SPG20* methylation has been demonstrated previously in several cancers [18, 19], including gastric cancer [20], we believe this is the first study to demonstrate *SPG20* methylation in IM, suggesting a role in early gastric carcinogenesis.

Infection by *H*. *pylori* is considered as a major risk factor for gastric cancer [21], capable of activating multiple signaling pathways including the JAK/STAT signaling [22, 23]. In this regard, activation of JAK/STAT signaling plays an important role in gastric carcinogenesis [24, 25]. However, the role of STAT3 in the epigenetic silencing of its targets is not fully elucidated. In this study, we found that the putative STAT3 target, *SPG20*, is epigenetically silenced in gastric cancer, similar to our previous findings of two other STAT3-epigenetically silenced targets, *NR4A3* [13] and *GATA3* [12]. Although a putative STAT3-binding site, as determined by ENCODE data, is few kb away from the promoter CpG island, the presence of the enhancer histone mark, H3K4me1, suggest that STAT3 might affect promoter methylation by higher-order chromatin structure. Such postulate is supported by the observation that Asian gastric cancer patients with more virulent and inflammatory-inducing strain of *H*. *pylori* strains [26–28] have higher *SPG20* methylation than non-Asian populations with less virulent strains (Fig 2E). However, further experiments are required to demonstrate the role of STAT3 in the epigenetic silencing of *SPG20*.

**Fig 4:**
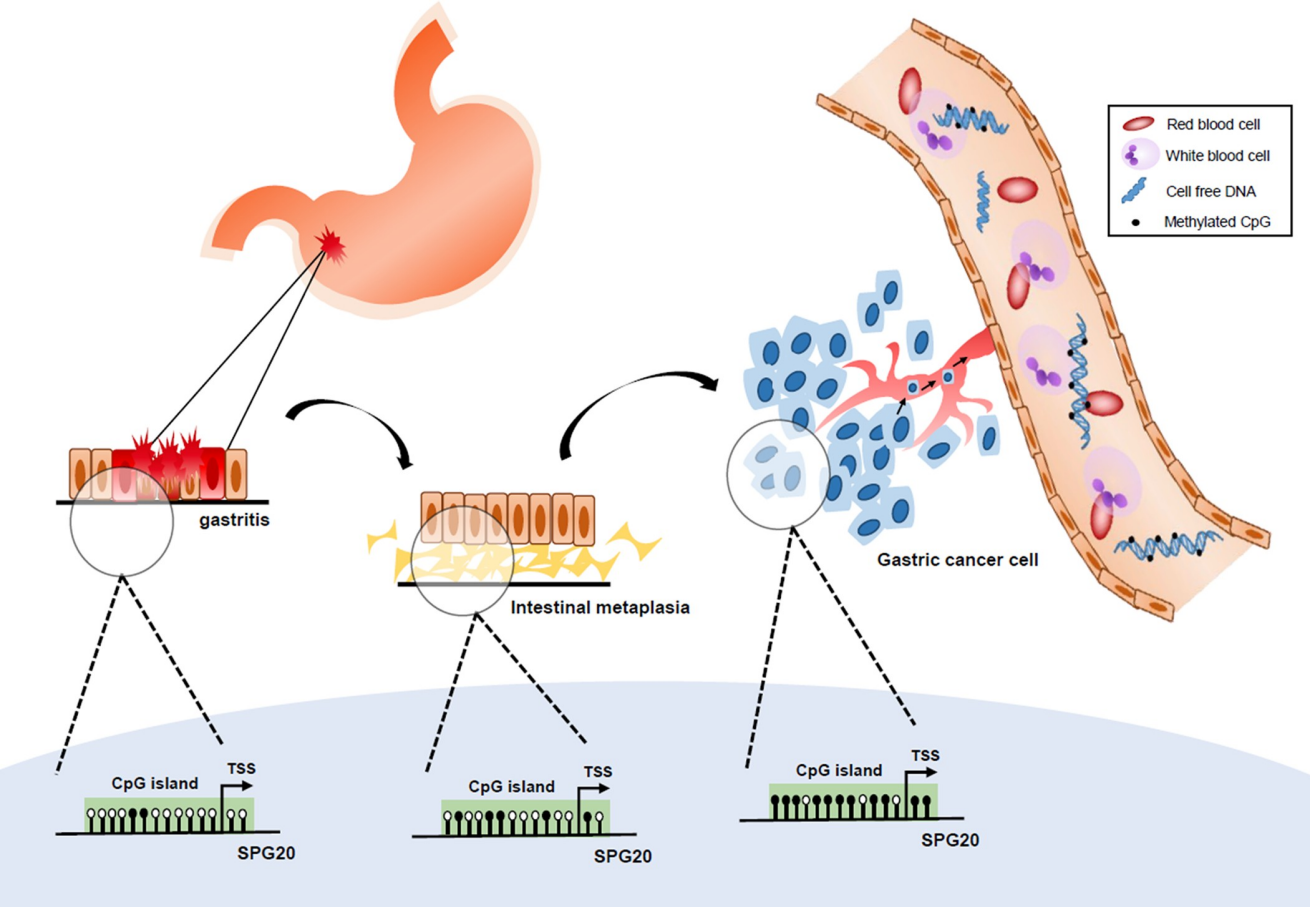
Schematic diagram showing a progressive increase of *SPG20* methylation in the development of gastric cancer. During the development of gastric cancer from gastritis to intestinal metaplasia to gastric cancer, a progressive increase in *SPG20* promoter methylation, probably due to increased activation of JAK/STAT signaling, is observed. Methylated DNA released into the peripheral blood as cell-free DNA, by necrosis or tumor metastasis, may then serve as a biomarker for the early detection of gastric cancer.

In this study, we also demonstrated that *SPG20* methylation could be detected in cell-free DNA isolated from serum samples of intestinal metaplasia (IM) and gastric cancer patients (Illustrated in Fig 4). Importantly, a progressive increase of *SPG20* methylation detection was observed from gastritis, to IM, and to cancer. However, more samples are required to determine the sensitivity and specificity of *SPG20* methylation in cancer diagnosis especially in the detection of early gastric cancer lesions.

In conclusion, we herein demonstrate that the putative STAT3 target, *SPG20*, is epigenetically silenced by promoter methylation in gastric cancer. and that such methylation is detectable in tissues and cfDNA from patients with gastric cancer as well as intestinal metaplasia. Consequently, *SPG20* methylation may be able to serve as a non-invasive biomarker for the early detection of gastric cancer.
